# Desarrollo físico y neuropsicológico de pacientes diagnosticados con hipotiroidismo congénito en el Hospital Universitario San Ignacio entre los años 2001 y 2017

**DOI:** 10.7705/biomedica.6334

**Published:** 2022-05-01

**Authors:** María Fernanda Unigarro, Catalina Forero, Camila Céspedes

**Affiliations:** 1 Departamento de Pediatría, Hospital Universitario San Ignacio, Bogotá, D.C., Colombia Departamento de Pediatría Hospital Universitario San Ignacio Bogotá D.C. Colombia; 2 Facultad de Medicina, Pontificia Universidad Javeriana, Bogotá, D.C., Colombia Pontificia Universidad Javeriana Facultad de Medicina Pontificia Universidad Javeriana Bogotá D.C. Colombia

**Keywords:** hipotiroidismo congénito, hormonas tiroideas, trastornos del neurodesarrollo, crecimiento, trastornos mentales, Congenital hypothyroidism, thyroid hormone, neurodevelopmental disorders, growth, thyroid hormones, mental disorders

## Abstract

**Introducción.:**

El hipotiroidismo congénito es la principal causa de discapacidad cognitiva prevenible en el mundo. Para detectarlo se han desarrollado programas de tamización, con el fin de disminuir las secuelas neurológicas asociadas. El seguimiento y las evaluaciones a mediano y largo plazo de estos pacientes son fundamentales.

**Objetivo.:**

Describir las características demográficas, el tratamiento y el seguimiento de los pacientes con diagnóstico de hipotiroidismo congénito en el marco del programa de tamización del Hospital Universitario de San Ignacio en Bogotá, Colombia.

**Materiales y métodos.:**

Se hizo un estudio observacional de corte transversal. La población de estudio fueron los pacientes con diagnóstico de hipotiroidismo congénito en el Hospital Universitario San Ignacio entre el 2001 y el 2017.

**Resultados.:**

Se contactó a 14 de los 19 pacientes con diagnóstico de hipotiroidismo congénito en el programa de tamizaje del Hospital. Los 14 niños estaban escolarizados, y la mayoría tenía el peso y la talla adecuados, aunque hubo talla baja en dos de ellos. El diagnóstico etiológico más frecuente fue hipoplasia tiroidea. Todos empezaron su tratamiento y el seguimiento oportunamente. La alteración más frecuente en las pruebas neuropsicológicas se registró en la memoria. El nivel de educación materno podría estar relacionado con el resultado anormal en el dominio del lenguaje.

**Conclusión.:**

En el presente estudio, las alteraciones en las pruebas de memoria fueron las más prevalentes; sin embargo, dado el diseño y el tipo de estudio, se requieren más investigaciones que permitan establecer asociaciones. El crecimiento y el desarrollo puberal presentaron una frecuencia baja de alteraciones.

## Abstract (en)

**Introduction:**

Congenital hypothyroidism is the leading cause of preventable cognitive disability in the world. Therefore, screening programs have been developed in order to reduce the neurological sequelae associated with this pathology.

**Objective:**

To describe the demographic characteristics, the treatment, and the follow-up of patients diagnosed with congenital hypothyroidism in the screening program at the San Ignacio University Hospital in Bogotá, Colombia.

**Materials and methods:**

We conducted an observational cross-sectional study. The study population was patients diagnosed with congenital hypothyroidism at the Hospital between 2001 and 2017.

**Results:**

Fourteen of the 19 patients diagnosed with congenital hypothyroidism in the hospital screening program were contacted. All of the patients had schooling, most of them had adequate weight and height, and two had short stature. In most of them, the etiological diagnosis was thyroid hypoplasia, and all began the treatment and follow-up in an adequate way. The most frequent alteration in the neuropsychological tests was in the memory domain and the level of maternal education could be related to an abnormal result in the domain of language.

**Conclusion:**

In our study, alterations in the memory tests were the most prevalent; however, due to the design and type of study, more research is required to establish associations. A low frequency of abnormal growth and puberty was found.

El hipotiroidismo congénito es la principal causa de discapacidad cognitiva prevenible. Por esta razón, a nivel mundial se han desarrollado programas de cribado, los cuales han sido fundamentales desde el punto de vista epidemiológico para disminuir las secuelas cognitivas asociadas con el hipotiroidismo no tratado. Esta es una condición que resulta de la disminución o ausencia de la actividad de las hormonas tiroideas en los tejidos, la cual comienza desde el nacimiento y puede ser secundaria a deficiencia en la producción o a resistencia frente a su acción ^1^^,^^2^.

Hay estudios que demuestran que el tratamiento de los pacientes a partir de las primeras dos a tres semanas de vida les permite tener un coeficiente intelectual normal, así como un crecimiento físico adecuado para su carga genética ^3^^-^^5^. En Colombia, se ha reportado una incidencia de uno de cada 2.183 nacidos vivos ^3^^,^^6^.

Los niños con hipotiroidismo congénito grave tienen alteraciones intelectuales significativas, comparados con la población general y con aquellos con hipotiroidismo moderado, pues sus puntajes cognitivos son más bajos, y presentan mayor inatención y menor desempeño verbal. También, se han observado menores logros en el aprendizaje, así como otras alteraciones del lenguaje y compromiso de la memoria autobiográfica ^6^^-^^8^.

En este contexto, el objetivo del presente estudio fue describir las características demográficas, el tratamiento y el seguimiento de los pacientes con hipotiroidismo congénito en el marco del programa de tamización del Hospital Universitario de San Ignacio en Bogotá, entre el 2001 y el 2017.

## Materiales y métodos

Se hizo un estudio observacional descriptivo y retrospectivo de la población de recién nacidos del Hospital con diagnóstico de hipotiroidismo congénito en el programa de tamización y que contaban con valoración actualizada de su desarrollo físico y su neurodesarrollo.

El protocolo contó con la aprobación del comité de investigaciones y ética institucional del Hospital. Esta fue la segunda fase de un estudio cuya primera etapa arrojó resultados que ya se encuentran publicados ^6^.

Se recolectaron los datos clínicos y demográficos mediante la revisión manual de las historias clínicas registradas en el sistema de administración de historias clínicas (SAHI). En los casos en los que la información fue insuficiente o no se encontraba disponible, esta se completó con los datos aportados por las familias en entrevistas telefónicas o virtuales, en las que también se explicó y se solicitó el diligenciamiento de los consentimientos y asentimientos informados para la realización de las pruebas neuropsicológicas, cuyos formatos se habían enviado por correo electrónico previamente.

En cuanto al seguimiento endocrinológico, se revisaron las historias clínicas, se compararon los resultados con el protocolo recomendado y se evaluó el cumplimiento de las citas de seguimiento. La gravedad del hipotiroidismo se calificó mediante los valores de T4L, según lo recomendado en la literatura especializada, como: grave, <0,38 ng/dl; moderado: 0,38 a 0,77 ng/dl, y leve, >0,77 ng/dl. Como parte del estudio etiológico, se evaluó la realización de ecografías y gammagrafías. En lo referente a las características del tratamiento, se recogió la información sobre este en el momento del diagnóstico y se preguntó a los pacientes o familiares la dosis diaria y por kg de peso formulada en el inicio del tratamiento, catalogándola como adecuada o no según las recomendaciones publicadas ^8^. En cuanto a la oportunidad del tratamiento, se reportó la edad de inicio, considerándola adecuada o inadecuada con base en el punto de corte de los 15 días de vida.

La emergencia de salud pública por la pandemia de SARS-CoV2 limitó los encuentros presenciales, por lo que la talla y el peso registrados en el momento de inicio del estudio correspondieron a los datos reportados en la historia clínica más reciente, siempre y cuando la valoración no excediera los tres meses. En la mayoría de los pacientes, este no era el caso, por lo que los familiares desde casa tomaron el peso y la talla guiados mediante llamadas telefónicas bajo la supervisión de una de las investigadoras del estudio, para garantizar el uso correcto de la técnica de medición.

El neurodesarrollo se valoró mediante pruebas neuropsicológicas específicas para cada rango de edad (5 a 6 años, 6 a 16 años y mayores de 16 años), para lo cual se solicitó el diligenciamiento del consentimiento y el asentimiento informados al padre o la madre y a los niños mayores de 8 años, respectivamente. Se exploraron las áreas de atención, lenguaje, memoria verbal y visual y funciones ejecutivas (cuadro 1). El resultado de estas pruebas se expresó como una variable dicotómica, tanto en lo global como en cada una de las esferas evaluadas, clasificándolas como adecuadas o inadecuadas según el puntaje obtenido por los participantes y el concepto de la neuropsicóloga infantil.

**Cuadro 1:** Pruebas neurocognitivas realizadas a cada grupo de edad

Grupo de edad	Atención	Lenguaje	Memoria	Funciones ejecutivas
5 a 6 años	Búsqueda de símbolos WPPSI	Vocabulario WPPSI III/	Curva ENI/figura de rey	Comprensión WPPSI III/
	III/retención de dígitos ENI	información WPSI III	ENI - recuerdo inmediato	reconocimiento de emociones ENI/ falsas creencias
6 a 16 años	Búsqueda de símbolos WISC	Vocabulario WISC IV/	Curva ENI/figura de rey	Comprensión WISC IV/reconocimiento de emociones ENI
	IV/retención de dígitos WISC IV	información WISC IV	ENI - recuerdo inmediato	
16 años en adelante	Búsqueda de símbolos WAIS III/ retención de dígitos WAIS III	Vocabulario WAIS III/ información WAIS III	Curva NEUROPSI/figura de rey - recuerdo inmediato	Comprensión WAIS II

WPPSI: Wechsler Preschool and Primary Scale of Intelligence

ENI: Evaluación Neuropsicológica Infantil

WISC: Wechsler Intelligence Scale for Children

NEUROPSI: Evaluación neuropsicológica breve en español

Se describieron las variables cuantitativas de edad, edad gestacional, peso, talla, dosis de inicio por kg, edad de inicio del tratamiento y talla parental, las cuales se expresaron en medidas de tendencia central y de dispersión (media y desviación estándar, DE). Las variables cualitativas se describieron mediante frecuencias y proporciones. Se utilizaron los programas Excel y Stata™, versión 12.0, para el análisis de los datos.

## Resultados

Se pudo contactar a 14 de los 19 pacientes con diagnóstico de hipotiroidismo congénito en el Hospital durante el periodo de estudio. De estos, nueve aceptaron participar en las pruebas neuropsicológicas.

La media de edad fue de 11,21 años (DE: ±3,94 años) y fueron 10 pacientes de sexo femenino y 4 de sexo masculino. La información sociodemográfica se presenta en el cuadro 2. Solo un paciente fue un recién nacido prematuro, de 32 semanas de gestación. El promedio del peso al nacer fue de 2.797,3 kg (DE: ±579,9 g); uno de los pacientes era pequeño para la edad gestacional y otro era grande.

**Cuadro 2:** Características sociodemográficas

Paciente	Sexo	Edad actual en años	Lugar de residencia	Régimen de salud
1	F	18	Urbano	Contributivo
2	F	17	Urbano	Contributivo
3	F	16	Urbano	Subsidiado
4	M	7	Urbano	Contributivo
5	M	14	Urbano	Contributivo
6	F	13	Urbano	Contributivo
7	F	10	Urbano	Contributivo
8	F	9	Urbano	Contributivo
9	F	8	Urbano	Contributivo
10	F	8	Urbano	Contributivo
11	M	8	Urbano	Contributivo
12	M	8	Rural	Contributivo
13	F	14	Urbano	Subsidiado
14	F	7	Urbano	Contributivo

En cuanto al peso y la talla, la mayoría de los pacientes tenía un adecuado desarrollo pondoestatural. Se encontró obesidad en tres pacientes, uno de ellos con antecedentes de macrosomía fetal. En uno de los pacientes con talla baja se estableció que su estatura estaba acorde con la talla medioparental. La clasificación de Tanner de todos los pacientes correspondía a la edad, aunque en el seguimiento de una paciente se describió telarquia a los 7 años y 8 meses, pero con una menarquia normal (cuadro 3).

**Cuadro 3:** Distribución por clasificación pondoestatural

Pacientes (N=14)		
IMC (kg/m2)		
	Normal	11
	Bajo peso	0
	Sobrepeso	0
	Obesidad	3
Talla		
	Normal	11
	Riesgo de baja talla	1
	Talla baja	2

**Figura 1:**
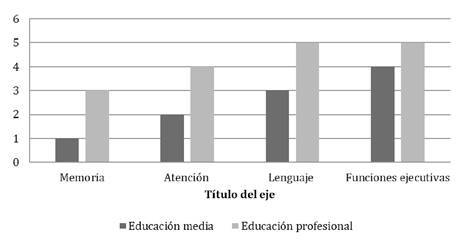
Distribución en números absolutos por nivel académico materno frente a resultado normal en las pruebas neuropsicológicas

Todos los pacientes estaban escolarizados, 28.57 % en primaria, 64.28 % en secundaria y 7.1 % en educación superior, solo uno no tenía el nivel de escolaridad adecuado para su edad, debido a ausentismo por razones familiares y no por mal rendimiento. En lo referente a la escolaridad de madres y padres, todos contaban con algún nivel de educación, aunque la mayoría de los padres no eran profesionales (57 %). En las figuras 1 y 2 se puede observar la distribución de los niveles educativos maternos frente a los resultados normales o anormales en cada dominio de la prueba neuropsicológica.

El promedio de edad de inicio del tratamiento fue a los 8 días de vida (DE: ±3,1 días). En los 14 pacientes analizados, el tratamiento se inició a la edad adecuada. El promedio de la dosis de inicio por kilogramo de peso fue de 10 μg/kg. Aunque un paciente recibió una dosis un poco menor que la ideal, debido a que la presentación del fármaco dificultaba su preparación y administración, todos los demás recibieron la dosis inicial adecuada. Todos los pacientes habían recibido tratamiento, por lo menos, hasta los siete años de edad, y dos lo suspendieron por indicación del endocrinólogo; de estos, uno continuó asistiendo a los controles y presentaba función tiroidea normal, aunque con un resultado global anormal en la prueba neuropsicológica y en los dominios de memoria y atención; sus progenitores tenían un nivel educativo básico: su padre hasta básica primaria y, su madre, educación media. La otra paciente no asistió a los controles después de la suspensión del tratamiento y su escolaridad era inadecuada debido a su ausentismo; además, no aceptó hacer la prueba neuropsicológica.

En cuanto a las comorbilidades, tres de los pacientes presentaban condiciones como estreñimiento, reflujo vesico-ureteral y rinitis alérgica. A 11 de los pacientes se les practicó ecografía de tiroides: en seis de ellos, arrojó resultados normales y, en los cinco restantes, se evidenció hipoplasia tiroidea. Solo dos pacientes tenían datos de gammagrafía de tiroides: en uno de ellos se confirmó la hipoplasia tiroidea y en el otro caso se diagnosticó bocio (cuadro 5).

**Figura 2:**
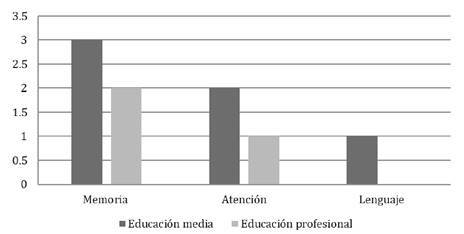
Distribución en números absolutos por nivel académico materno frente a resultado anormal en las pruebas neuropsicológicas

**Cuadro 5:** Resultados de la ecografía de tiroides y la gammagrafía

Paciente (N=14)		
Ecografía de tiroides		11
	Normal	6
	Anormal (hipoplasia tiroidea)	5
Gammagrafía de tiroides		2
	Hipoplasia	1
	Bocio	1

Con la información recolectada, se pudo establecer que tres de los pacientes habían asistido a su primer control después del inicio oportuno del tratamiento (según los criterios de la *American Academy of Pediatrics* - AAP) ^8^, y que, por lo menos, la primera consulta después del inicio del tratamiento se había hecho en el Hospital Universitario San Ignacio. Sin embargo, solo dos pacientes continuaron sus controles en este hospital: una de ellas tuvo seguimiento durante sus primeros cuatro años de vida, con consultas en los intervalos recomendados, pero después de esta edad, cambió de lugar de seguimiento. Durante sus controles en el Hospital, se constató un adecuado neurodesarrollo; actualmente, la paciente sufre de sobrepeso, tiene una escolaridad acorde para la edad y obtuvo un puntaje normal en todos los dominios de la prueba neuropsicológica. La otra paciente aún continúa asistiendo a sus citas de control en el Hospital y presentó retraso del neurodesarrollo, con compromiso de la motricidad gruesa y el lenguaje, por lo cual requirió seguimiento por neuropediatría, así como terapia física y del lenguaje en el primer año de vida; tuvo una adecuada evolución y su resultado global en la prueba neuropsicológica fue normal.

En cuanto a los hitos del desarrollo, la mayoría de los pacientes los alcanzaron oportunamente según lo referido por los padres. Solo se detectó un paciente que había recibido tratamiento en el primer año de vida, con retraso en el neurodesarrollo y compromiso de la motricidad gruesa y el lenguaje.

De los 14 pacientes que se contactaron, nueve aceptaron someterse a la prueba neuropsicológica, cuyos resultados se presentan en el cuadro 6. La mayoría de los pacientes tuvo resultados anormales en la prueba debido a alteraciones en alguno de los dominios valorados. Solo un paciente presentó alteraciones en todos los dominios, excepto en el de las funciones ejecutivas, por lo que fue remitido para valoración por neuropediatría y se sugirió hacerle la prueba de inteligencia. Los demás pacientes solo tuvieron alteraciones en uno de los ítems evaluados. El mayor número de alteraciones se presentó en el dominio de la memoria; en la valoración clínica de la neuropsicóloga se sugirió una evaluación neuropsicológica completa de los cinco pacientes con baja puntuación en memoria, y una prueba cognitiva para los cuatro con alteraciones de la atención. De los pacientes analizados, solo uno tuvo resultados anormales en la prueba de lenguaje y ninguno tuvo alteraciones en el dominio de las funciones ejecutivas.

## Discusión

El hipotiroidismo congénito no tratado es la causa más común de retraso mental, el cual se puede prevenir mediante un tratamiento temprano ^3^^,^^7^^,^^9^. En algunos estudios de cohorte, se ha descrito que los niños con hipotiroidismo congénito grave incluso con tratamiento pueden presentar alteraciones neurocognitivas, con puntajes menores en las pruebas de neurodesarrollo, alteraciones de la atención y retraso en el desarrollo verbal, comparados con los niños sin hipotiroidismo ^9^^,^^10^.

En otros estudios, como el de Baysal, *et al*., se concluyó que el estado de desarrollo neurológico de pacientes con hipotiroidismo congénito tratado no fue significativamente diferente al de sus compañeros no afectados ^11^. En el presente estudio, la evaluación de los hitos de desarrollo de la mayoría de ellos se hizo en la entrevista con los padres, no con base en la historia clínica, y se encontró que en 13 de los 14 pacientes reportaban un neurodesarrollo normal, aunque pudo haber un sesgo de memoria en dicha valoración. Sin embargo, si se presentaba alguna alteración, esta debió ser sutil, pues, en general, los padres recuerdan los problemas mayores.

**Cuadro 6:** Distribución por resultado de la prueba neuropsicológica global y por dominios

Pacientes (N=14)		
Prueba neuropsicológica		
	Normal	3
	Anormal	6
Prueba de memoria		
	Anormal	4
	Normal	5
Prueba de memoria verbal auditiva		
	Normal	6
	Anormal	3
Prueba de memoria visual		
	Normal	8
	Anormal	1
Prueba de lenguaje		
	Normal	8
	Anormal	1
Prueba de atención		
	Normal	5
	Anormal	4
Prueba de atención sostenida		
	Normal	6
	Anormal	3
Prueba de memoria de trabajo		
	Normal	5
	Anormal	4
Prueba de volumen de retención		
	Normal	5
	Anormal	4
Prueba de funciones ejecutivas		
	Normal	9
	Anormal	0

En otro estudio, Komur, *et al*., señalaron que ni la dosis ni el día de inicio del tratamiento con medicamentos de suplencia para el hipotiroidismo, ni la gravedad de la enfermedad o el tiempo de normalización de la hormona estimulante de la tiroides, tuvieron efectos estadísticamente significativos sobre el desarrollo neurológico del grupo de estudio (p>0,05) ^10^. En el presente estudio, se encontró un caso de dificultad para alcanzar los hitos del desarrollo que requirió terapia de apoyo durante el primer año, a pesar de que la paciente recibía el tratamiento con levotiroxina en la dosis adecuada y el tiempo recomendado; cuando se revisó la evolución del perfil tiroideo se encontró que se había requerido un mes para normalizar las pruebas ^10^. Esta paciente hoy tiene un excelente rendimiento escolar y su prueba neuropsicológica fue normal.

Se ha informado que el hipotiroidismo congénito está asociado con un deterioro del desarrollo neurológico en pacientes diagnosticados en el marco de programas de cribado neonatal ^12^. En este estudio, se encontró que, de los seis pacientes con pruebas neuropsicológicas anormales, tres tenían hipotiroidismo leve en el momento del diagnóstico, dos, hipotiroidismo moderado, y uno, hipotiroidismo grave (gravedad establecida por los niveles de T4L: grave, <0,38 ng/dl; moderado, 0,38 a 0,77 ng/dl, y leve, >0,77) ^12^. Se encontró que los niños con hipotiroidismo congénito presentaron algunas alteraciones en las pruebas específicas de memoria verbal, memoria auditiva y atención, a pesar de que todos iniciaron el tratamiento con las dosis recomendadas antes de los 15 días de vida. En aquellos que hicieron las pruebas neuropsicológicas, hubo alteraciones en varias de las áreas evaluadas.

El paciente con hipotiroidismo grave en el momento del diagnóstico tuvo que ser remitido a neuropediatría y se le hizo la prueba de inteligencia, ya que tenía puntajes muy bajos en todos los dominios, excepto en el de las funciones ejecutivas. No se contó con el seguimiento de la función tiroidea de este paciente, pero el tiempo y la dosis de inicio del tratamiento fueron adecuados y aún recibe suplencia hormonal con 50 μg diarios de levotiroxina. Esto concuerda con lo descrito en la literatura con respecto al impacto de la gravedad del hipotiroidismo al nacer, a pesar del diagnóstico y el tratamiento tempranos.

En cuanto a la prueba de lenguaje, en un estudio del 2020 de Lamônica, *et al*., se compara el desempeño de las habilidades motoras gruesas, la motricidad fina adaptativa, la lingüística, la cognitiva, y el desarrollo personal y social de niños con hipotiroidismo congénito tratados desde el período neonatal, con el de sus pares sin alteraciones tiroideas. Los autores concluyen que, al comparar los resultados de las pruebas de detección del desarrollo de Denver 2, se encontró una diferencia estadísticamente significativa entre los grupos en las áreas de lenguaje y motricidad fina y gruesa. En el presente estudio, se describe la alteración en las pruebas de lenguaje de uno de los pacientes, que tenía hipotiroidismo grave en el diagnóstico ^13^.

En otro estudio se señala que, a medida que aumenta el nivel de educación de las madres, también aumentan las puntuaciones de desarrollo del lenguaje, y nuestros resultados sugieren algo similar: de los tres pacientes con hipotiroidismo congénito grave, se pudo contactar a dos, cuyos resultados en la prueba neuropsicológica fueron: uno anormal, incluso en el dominio de lenguaje, y el otro con una prueba normal en todos los dominios. La madre del paciente con resultados anormales, incluso en el dominio del lenguaje, tenía un nivel de educación medio, en tanto que la madre del paciente con prueba normal tenía educación superior ^10^^,^^11^.

Se ha descrito que los niños con bajas concentraciones de TSH presentan disminución de la atención sostenida en la edad escolar ^4^. Por nuestra parte, pudimos establecer que los pacientes que tuvieron un seguimiento oportuno y un adecuado control de la enfermedad no presentaron alteraciones en las pruebas neuropsicológicas; sin embargo, no fue posible establecer la variabilidad de los valores séricos de TSH y T4l con el tratamiento durante el seguimiento, pues no se contaba con todos los datos, lo que resalta la necesidad y la pertinencia de centralizar el seguimiento de estos pacientes.

En su estudio, Dalili, *et al*., compararon el crecimiento y el desarrollo de los pacientes con hipotiroidismo congénito y el de niños sanos de la misma edad, área geográfica, y clase social y económica a los cuatro años (ajustado por variables sociodemográficas). Las características demográficas, incluida la altura, el peso y la circunferencia de la cabeza al nacer, el tiempo de seguimiento (a cuatro años) y el coeficiente intelectual, se registraron mediante diferentes pruebas. Entre 28.904 recién nacidos examinados, se encontraron 37 pacientes con hipotiroidismo congénito. Los pacientes diagnosticados mediante cribado neonatal y tratados tuvieron un crecimiento normal, como el de la población general. No obstante, los autores señalan que probablemente no se encontraron diferencias significativas debido al tamaño de la muestra ^2^.

En ese orden de ideas, en el presente estudio, el peso, la talla y el índice de masa corporal (IMC) de la mayoría de los pacientes fueron normales. Un pequeño porcentaje presentaba riesgo de talla baja o talla baja, sin embargo, por lo menos, uno de los casos podría corresponder a talla baja familiar, aunque, lamentablemente, no hubo seguimiento endocrinológico de estos pacientes a lo largo del tiempo, lo que dificulta la interpretación de nuestros resultados.

En cuanto al diagnóstico etiológico de los pacientes que tenían resultados de exámenes complementarios, no se encontró ninguno con diagnóstico de ectopia, a pesar de que esta es la causa más frecuente de hipotiroidismo congénito ^2^.

Como ya se ha comentado, es clara la necesidad de centralizar el seguimiento de estos pacientes para establecer de una forma más precisa el impacto del programa de tamizaje neonatal en todos sus aspectos (detección, tratamiento, seguimiento).
